# Western New York Association

**Published:** 1897-11

**Authors:** 


### WESTERN NEW YORK ASSOCIATION.

The Western New York Homœopathic Medical Association held a meeting at Lockport, N. Y., on Friday, October 15th. The meeting was presided over by Dr. W. H. Hodge, of Niagara Falls. Nineteen papers were read by their authors before the meeting, which included seventy members. Among these papers may be mentioned "Progressive Myopia," by Dr. E. J. Bissell, of Rochester; "The Effects of Alcohol on the System," by Dr. Louise J. Chamberlayne, of Rochester; "Preventive Measures for Various Diseases," by Dr. N. M. Collins, Rochester; "The Therapeutics of the Old School," by Dr. W. B. Gifford, of Attica; "Notions," by Dr. T. J. Greenleaf, Superintendent of the Glen Mary Home, at Owego; "Hay Fever," by Dr. Julia F. Haywood, of Rochester; "Cancer of the Uterus from the Standpoint of the General Practitioner," by Dr. J. M. Lee, of Rochester; "The Physician as a Business Man," by Dr. J. W. Le Seur; "Artificial Light in Its Effects Upon the Eyes," by Dr. F. Park Lewis, of Buffalo; "Some Methods of Treating Fractures of the Thigh," by Dr. G. T. Moseley, of Buffalo; "An Experience with Chloroform," by Dr. Joseph Reiger, of Dunkirk. There were many other papers equally worthy of notice, but those given above show the general character of the proceedings.

